# Microbial yield from infectious tissues pretreated by various methods: an invitro study

**DOI:** 10.1186/s12891-021-04071-5

**Published:** 2021-02-21

**Authors:** Yuanqing Cai, Xinyu Fang, Lvheng Zhang, Xurong Yang, Lixiong Nie, Zida Huang, Wenbo Li, Chaofan Zhang, Bin Yang, Zhenpeng Guan, Wenming Zhang

**Affiliations:** 1 Department of Orthopaedic Surgery, The First Affiliated Hospital of Fujian Medical University, Fuzhou, China; 2 Department of Laboratory Medicine, The First Affiliated Hospital of Fujian Medical University, Fuzhou, China; 3 Department of Orthopedic Surgery, Peking University Shougang Hospital, Beijing, China

**Keywords:** Pretreatment methods, Tissues, Microbial recovery, Periprosthetic joint infection, Periprosthetic tissues

## Abstract

**Background:**

This study aimed to evaluate the effects of different pretreatment methods on the microbial yield from infectious tissues.

**Methods:**

Strains of *Staphylococcus aureus* (SA), *Escherichia coli* (EC) and *Candida albicans* (CA) were used to construct single-surface, full-surface, and internal infection models in sterile pork tissue. Manual milling (MM), mechanical homogenization (MH), sonificated (SF), dithiothreitol (DTT), and direct culture (DC) were used to pretreat these tissues, the microbial yield from different pretreatment methods were recorded and compared. Moreover, periprosthetic tissues collected intraoperatively from periprosthetic joint infection (PJI) patients were used as a verification.

**Results:**

The study showed that the microbial yield from MH pretreatment was significantly higher than that of MM (*P* < 0.01) and SF pretreatment method (*P* < 0.01). Furthermore, in the internal infection model, the microbial yield from MH group was also significantly higher than that of SF (*P* < 0.01), DTT (*P* < 0.01), and DC group (*P* < 0.01). Moreover, the number of bacterial colonies obtained from periprosthetic tissues pretreated by MH was significantly higher than pretreated by other pretreatment methods (*P* = 0.004).

**Conclusions:**

The effects of MH and DTT in microbial yield were significantly higher than that of DC, SF and MM, and these methods can be used to process multiple tissue samples at the same time, which might further improve the diagnostic sensitivity of infectious disease.

**Supplementary Information:**

The online version contains supplementary material available at 10.1186/s12891-021-04071-5.

## Background

Periprosthetic joint infection (PJI) is a serious complication after joint arthroplasty, which brings a heavy economic burden to patients and society [1]. Microbial culture is essential for the diagnosis and treatment of PJI [2, 3]. At present, the laboratory diagnostic methods mainly based on synovial fluid, sonication fluid and periprosthetic tissues culture, but in many cases, synovial/sonication fluid are insufficient. However, It is feasible to obtain sufficient tissue samples intraoperatively, but the positive microbial culture rates are frustrating if tissues are not properly pretreated [4]. Therefore, optimizing the pretreatment methods of tissue specimens to make full use of PJI tissue samples is of great significance for improving the positive culture rate.

The current mainstream tissue pretreatment methods reported in clinical microbiology laboratories are direct tissue culture (DC) and manual milling (MM) [5]. However, MM is a tedious process in which contamination can easily be introduced. Studies have shown that mechanical homogenization (MH), dithiothreitol (DTT), and sonificated (SF) can increase the release of bacteria from tissue [6–8]. However, wether these methods are clinical available are still in the air, because currently there are no reports that directly compared these different specimen pretreatment methods in vitro.

In this study, the abovementioned pretreatment methods were used to pretreat infectious tissues models constructed in vitro and tissue specimens collected from PJIs. The recovery colony-forming units (CFU) were recorded and compared to evaluate the efficiency of different pretreatment methods.

## Methods

This study was approved by institutional review board. For PJI cases, the demographic data, medical history, laboratory tests and microbial culture results were prospective collected from January 2018 to December 2019 in our center, and all patients signed informed consent forms. PJI was diagnosed based on the American Society for Musculoskeletal Infection (MSIS) criteria for PJI.

### Establishment of infection tissues model

Fresh pork was frozen and cut into cubes at a size of 0.5 × 0.5 × 0.5 cm^3^, each weighing approximately 40 mg. After immersion in diluted penicillin G (500 U/ml), streptomycin (600 μg/ml) and amphotericin B (2.5 μg/ml) for 1 h, the samples were washed three times with PBS.

**Fig. 1:**
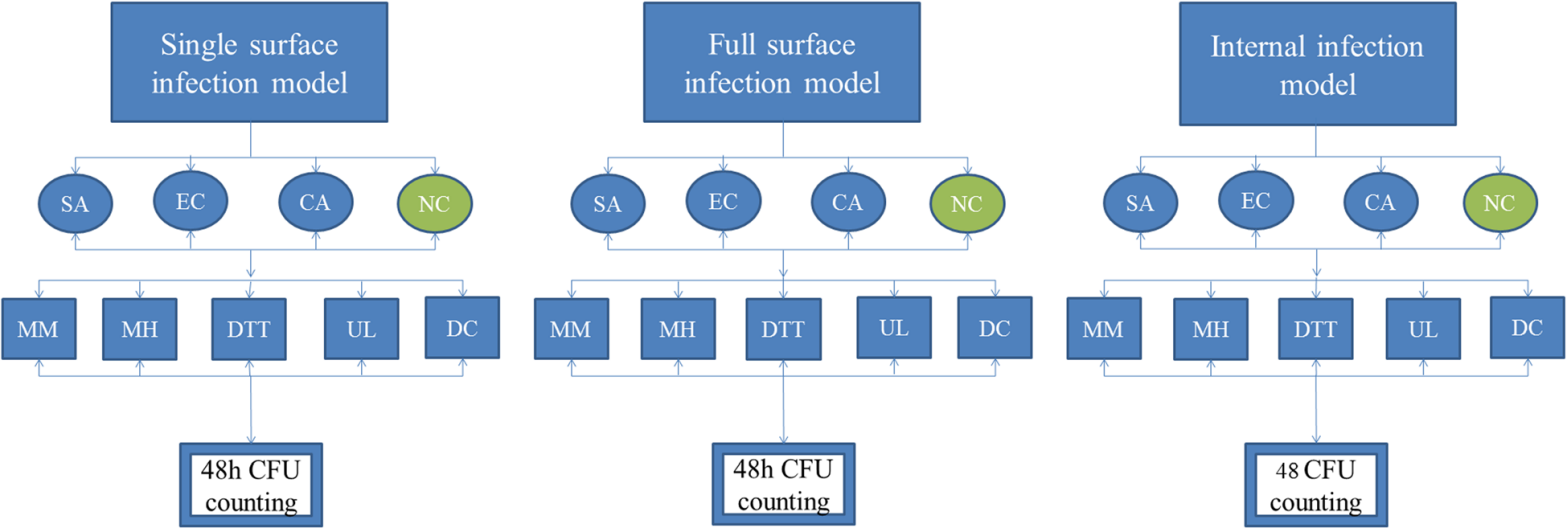
Establishment of infection tissues in vitro

The strain of *Staphylococcus aureus* (*S. aureus*) ATCC 25923 (American Type Culture Collection, USA) the strain of *Escherichia coli* (*E. coli*) ATCC 25922 (American Type Culture Collection, USA), and the strain of *Candida albicans* (*C. albicans*)ATCC 90029 (American Type Culture Collection, USA) were used to establish infection model in vitro. In order to simulate PJI infection as much as possible, in which pathogen may be distributed on a single surface or multiple surfaces even inside tissues [6], therefore, three tissues infection models were established (Fig. 1): ① Single surface infection: pipette aspirate 10 μL (200 CFU) bacterial dilution (*S. aureus /E. coli/Candida albicans*) to colonized a single surface of the pork cube. ② Full surface infection model: Microorganisms were colonized on the entire surface of pork cubes, with 10 μL (200 CFU) for each surface. ③ Internal infection model: Ten microliters (200 CFU) of bacterial dilution was injected into the center of the pork cube with a syringe for colonization. And the control group which have an incision on the surfaces of the pork was injected with the same amount of saline, to simulated a surgical wound was also established. Fifty pieces of each pathogen model were made, and the pork samples were inoculated for an hour.

### Various pretreatment methods on infectious tissues model in vitro

Fifty samples of each infection model were randomly assigned to MH group, MM group, DTT group, SF group, and DC group. For pretreatment: ① MH group: tissues were transferred to 2 mL Eppendorf (EP) tubes with 1 mL LB culture medium (Haibo Biotechnology Co., Ltd., Qingdao, Chian), vortexed for 15 min, and put into a fully automatic rapid grinder (Jingxin Industrial Development Co., Ltd., Shanghai, China) at 40 Hz for 60–90 s until the tissue specimen was homogenized. ② MM group: tissues were transferred to 2 mL EP tubes with 1 mL LB culture medium and manually ground with a disposable sterile grinding rod until a homogenate appeared. ③ DTT group: the shock time and DTT concentration established in previous in vitro studies was used [9]. After soaking in 1 g/L DTT (Haibo Biotechnology Co., Ltd., Qingdao, China) for 15 min at room temperature, specimens were transferred to a 2 mL EP tube and 1 mL of LB liquid medium was added to seal the tubes. ④ SF group: tissues were transferred to 2 mL EP tubes with 1 mL LB culture medium, vortexed and shaken for 30 s and placed in an ultrasonic cleaner (Wuxi Woxin Instrument Co., Ltd., Jiangsu, China), sonicated at 40 Hz for 5 min. ⑤ DC group: tissues were transferred to 2 mL EP tubes with 1 mL LB culture medium and vortexed for 15 min.

After pretreatment, the inoculated specimens were centrifuged at 5500 rpm/min after the above four tissue pretreatment methods for 20 s to spin down impurities. After centrifugation, 100 μL of each solution was inoculated on Columbia blood agar plates (Thermo Fisher Scientific, USA) and CDC anaerobic blood agar plates (Qingdao Haibo Biotechnology Co., Ltd., HBPM16) under aerobic and anaerobic conditions, respectively, overnight at 37 °C in a biochemical incubator (Shanghai Qixin Scientific Instrument Co., Ltd., China). Finally, CFUs were obtained and calculated.

### Various tissues pretreatment methods on periprosthetic tissues collected from PJIs

A total of 30 tissue specimens were collected from 8 PJI patients who were diagnosed according to MSIS criteria for PJI [10]. Each tissue specimen was divided into 5 equal portions (each = 50 mg) and treated with the above methods (MH, MM, DTT, SF, DC).

One hundred microliters of each of the inoculated samples was inoculated onto Columbia blood agar plates (Thermo Fisher Scientific, USA) and CDC anaerobic blood agar plates (Qingdao Haibo Biotechnology Co., Ltd., HBPM16) under aerobic and anaerobic conditions, respectively. Samples were incubated in a biochemical incubator (Shanghai Qixin Scientific Instrument Co., Ltd., China) at37 °C for 14 days, and colony growth was observed 2, 4, 7, and 14 days after incubation. If there was colony growth, the CFU per ml were calculated.

### Statistical analysis

The continuous variables were expressed as mean ± standard deviation. One- way ANOVA and post-test were used to compared the differences between groups. All statistical analysis was performed on GraphPad Prism 8 .0. *P* < 0.05 was regarded as statistically significance.

## Results

### Microbial yield recovered from single-surface infection models pretreated by various methods

**Fig. 2:**
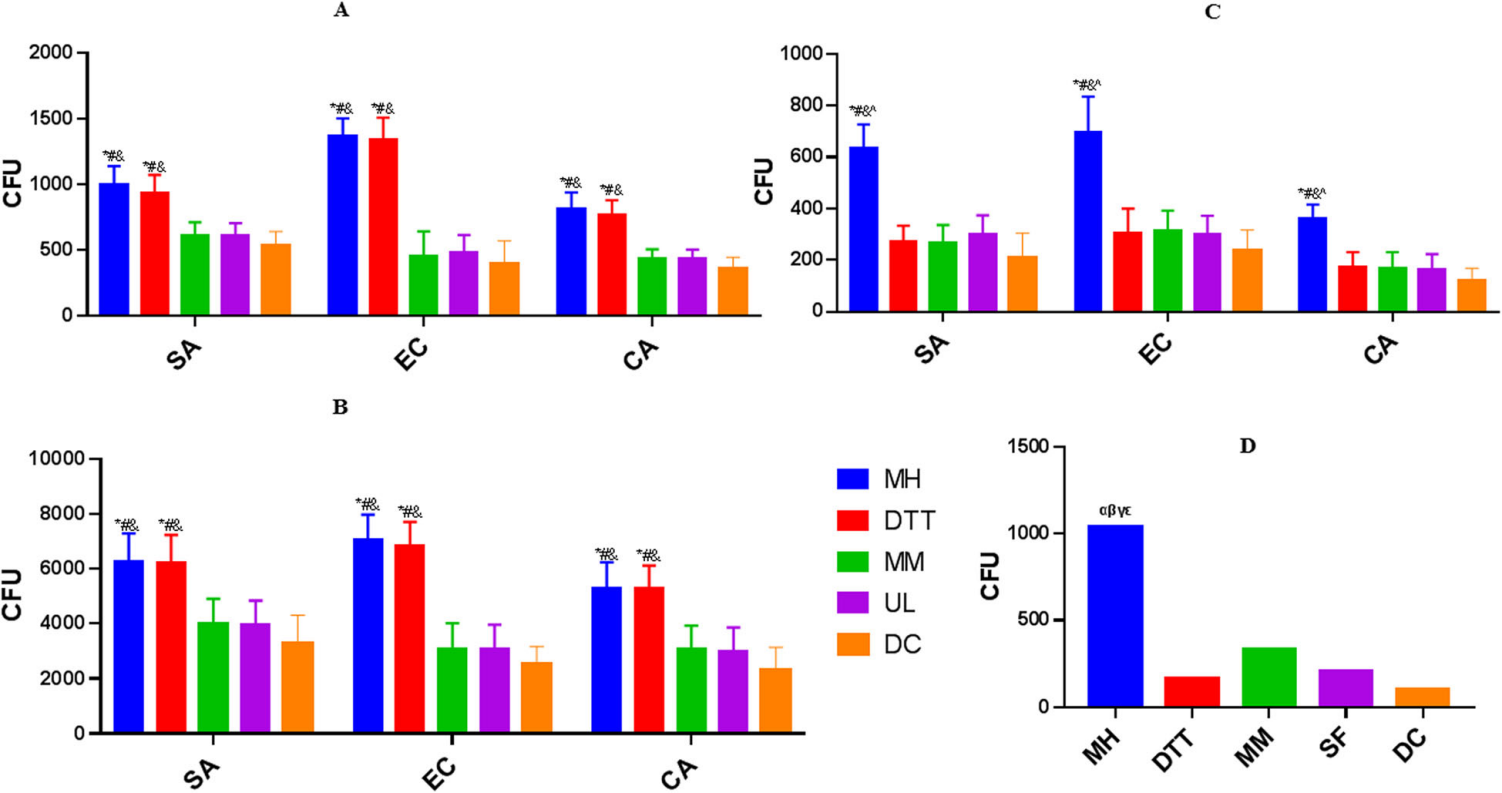
Quantitative recovery of microorganisms from inoculated tissues. Tissue cubes were separately inoculated on 1 surface (**a**), on six surfaces (**b**), or on the inside of the samples (**c**) with 2 × 102 CFU of *S. aureus, E. coli, or C. albicans,* respectively. Number of colony forming units (CFU) obtained from PJI patient specimens by various pretreatment methods (**d**).CFU = colony forming units; *P < 0.01 compared to the DTT group, #*P* < 0.01 compared to the MM group, & *P* < 0.01 compared to the SF group, ^*P* < 0.01 compared to the DC group. α *P* < 0.05 compared to the DTT group, β *P* < 0.05 compared to the MM group, γ P < 0.05 compared to the SF group, ε *P* < 0.05 compared to the DC group

In the SA infection groups, the average CFU of the MM, MH, SF, DTT, and DC groups were 611 ± 101 CFU/ml (95% CI: 538–683), 999 ± 141 CFU/ml (95% CI: 898–1100), 609 ± 96 CFU/ml (95% CI: 540–679), 938 ± 136 CFU/ml (95% CI: 840–1035), and 533 ± 108 CFU/ml (95% CI: 455–611), respectively. The CFUs obtained in MH group was significantly higher than that in the MM (*P* < 0.01), SC (*P* < 0.01), and SF groups (*P* < 0.01), but there was no significant difference with that of the DTT group (*P* = 0.26) (Supplementary file; Fig. 2a).

In the EC infection groups, the average CFU of the MM, MH, SF, DTT, and DC groups were 451 ± 192 CFU/ml (95% CI: 313–588), 1373 ± 132 CFU/ml (95% CI: 1278–1468), 480 ± 135 CFU/ml (95% CI: 383–576), 1347 ± 162 CFU/ml (95% CI: 1231–1463), and 393 ± 178 CFU/ml (95% CI: 265–520), respectively. The CFUs obtained in the MH group was significantly higher than that in the MM (*P* < 0.01), SF (*P* < 0.01), and DC groups (*P* < 0.01), but there was no significant difference from that of the DTT group (*P* = 0.71). (Supplementary file; Fig. 2a).

In the CA infection groups, the average CFU of the MM, MH, SF, DTT, and DC groups were 440 ± 70 CFU/ml (95% CI: 391–488), 818 ± 122 CFU/ml (95% CI: 730–905), 435 ± 68 CFU/ml (95% CI: 386–484), 771 ± 108 CFU/ml (95% CI: 694–849), and 360 ± 85 CFU/ml (95% CI: 298–421), respectively. The CFUs obtained in the MH group was significantly higher than that in the MM (*P* < 0.01), SF (*P* < 0.01) and DC groups (*P* < 0.01), but there was no significant difference with that of the DTT group (*P* = 0.945). (Supplementary file; Fig. 2a).

### Microbial yield recovered from full-surface infection models pretreated by various methods

In the SA infection groups, the average CFU of the MM, MH, SF, DTT, and DC groups were 4014 ± 888 CFU/ml (95% CI: 3378–4650), 6268 ± 1019 CFU/ml (95% CI: 5539–6997), 3960 ± 875 (95% CI: 3334–4568) CFU/ml, 6227 ± 1000 CFU/ml (95% CI: 5512–6942), and 3255 ± 1048 CFU/ml (95% CI: 2505–4005), respectively. The CFUs obtained in the MH group was significantly higher than that in the MM (*P* < 0.01), SF (*P* < 0.01) and DC groups (*P* < 0.01), but there was no significant difference compared with that of the DTT group (*P* = 0.924). (Supplementary file; Fig. 2b).

In the EC infection groups, the average CFU of the MM, MH, SF, DTT, and DC groups were 3079 ± 934 CFU/ml (95% CI: 2410–3747), 7058 ± 920 CFU/ml (95% CI: 6400–7717), 3060 ± 902 CFU/ml (95% CI: 2415–3706), 6853 ± 858 CFU/ml (95% CI: 6239–7466), and 2533 ± 637 CFU/ml (95% CI: 2077–2989), respectively. The CFUs obtained in the MH group was significantly higher than that in the MM (*P* < 0.01), the SF (*P* < 0.01), and DC groups (*P* < 0.01), but there was no significant difference compared with that of the DTT group (*P* = 0.964). (Supplementary file; Fig. 2b).

In the CA infection groups, the average CFU of the MM, MH, SF, DTT, and DC groups were 3080 ± 842 CFU/ml (95% CI: 2477–3682), 5274 ± 966 CFU/ml (95% CI: 4583–5966), 2971 ± 890 CFU/ml (95% CI: 2334–3608), 5269 ± 853 CFU/ml (95% CI: 4658–5879), and 2287 ± 851 CFU/ml (95% CI: 1678–2896), respectively. The CFUs obtained in the MH group was significantly higher than that in the MM (*P* < 0.01), SF (*P* < 0.01), and DC groups (*P* < 0.01), but there was no significant difference compared with that of the DTT group (*P* = 0.989). (Supplementary file; Fig. 2b).

### Microbial yield recovered from internal infection models pretreated by various methods

In the SA infection groups, the average CFU of the MM, MH, SF, DTT, and DC groups were 267 ± 68 CFU/ml (95% CI: 218–316), 636 ± 90 CFU/ml (95% CI: 571–701), 300 ± 73 CFU/ml (95% CI: 247–353), 270 ± 63 CFU/ml (95% CI: 224–315), and 208 ± 95 CFU/ml (95% CI: 140–277), respectively. The CFUs obtained in the MH group was significantly higher than that in the MM (*P* < 0.01), SC (*P* < 0.01), DTT (*P* < 0.01), and SF groups (*P* < 0.01). (Supplementary file; Fig. 2c).

In the EC infection groups, the average CFU of the MM, MH, SF, DTT, and DC groups were 313 ± 78 CFU/ml (95% CI: 257–369), 698 ± 134 CFU/ml (95% CI: 601–794), 298 ± 72 CFU/ml (95% CI: 246–351), 306 ± 93 CFU/ml (95% CI: 239–373), and 234 ± 82 CFU/ml (95% CI: 175–293), respectively. The CFUs obtained in the MH group was significantly higher than that in the MM group (*P* < 0.01), SF group (*P* < 0.01), DTT group (*P* < 0.01), and DC group (*P* < 0.01). (Supplementary file; Fig. 2c).

In the CA infection groups, the average CFU of the MM, MH, SF, DTT, and DC groups were 169 ± 60 CFU/ml (95% CI: 125–212), 359 ± 55 CFU/ml (95% CI: 319–399), 164 ± 58 CFU/ml (95% CI: 122–206), 172 ± 58 CFU/ml (95% CI: 130–213), and 119 ± 47 CFU/ml (95% CI: 85–154), respectively. The CFUs obtained in the MH group was significantly higher than that in the MM group (*P* < 0.01), SF group (*P* < 0.01), DTT group (*P* < 0.01), and DC group (*P* < 0.01). (Supplementary file; Fig. 2c).

### Microbial culture results of control samples

Negative (sterile) control samples consisted of 30 cubes of pork tissue (10 for each single-surface/full-surface/internal model). In the MM group, *Streptococcus viridans* and *Staphylococcus epidermidis* were isolated from negative control of single-surface and full-surface models, respectively. And *Corynebacterium*, which was considered as a contaminant, was isolated from negative control pretreated by SF (Table 1).

**Table 1:** Microbial culture results of control samples

	MH	MM	DTT	SF	DC
Single surface infection model	0/10	1/10 *Streptococcus viridans* (10 CFU)	0/10	0/10	0/10
Full surface infection model	0/10	1/10 *Staphylococcus epidermidis* (20 CFU)	0/10	0/10	0/10
Internal infection model	0/10	0/10	0/10	1/10 Corynebacterium (20 CFU)	0/10

### Microbial yield of tissue samples collected from PJIs intraoperatively pretreated by various methods

The numbers of CFUs obtained from tissues collected from PJIs subjected to various pretreatment methods were listed in Table 2. Among 30 tissue samples, from 8 PJI patients, 10 tissue samples showed at least one positive microbial culture result, which collected from 7 cases. Only one patient’s microbial culture results were all negative from tissues samples pretreated by various methods. The total number of CFUs obtained by MH was significantly higher than that obtained by the other pretreatment methods (Fig. 2d, *P* = 0.004).

**Table 2:** Microbial yield of tissue samples collected from PJIs intraoperatively pretreated by various methods

Original specimen	Pathogenic bacteria	MH	MM	DTT	SF	DC
1 (case 1)	*Staphylococcus epidermidis*	10	0	0	1	0
2 (case 2)	*Staphylococcus aureus*	360	50	179	60	34
3 (case 3)	*E.coli*	187	23	45	8	10
4 (case 3)	*E.coli*	68	40	20	23	30
5 (case 5)	*Staphylococcus epidermidis*	96	22	36	28	16
6 (case 6)	*Enterococcus faecalis*	46	0	33	0	0
7 (case 6)	*Enterococcus faecalis*	89	17	46	34	0
8 (case 7)	*Staphylococcus epidermidis*	147	0	36	28	0
9 (case 7)	*Staphylococcus epidermidis*	15	0	0	0	0
10 (case 8)	*Staphylococcus aureus*	283	12	16	26	10
Total		1041	164	329	208	100

## Discussion

In the present work, various infection tissues models were established in vitro, together with tissues collected from PJI patients, were pretreated by different methods, finally the microbial yield were recorded and compared. This study showed that the effects of MH and DTT in microbial yield from tissues were significantly higher than that of DC, SF and MM, and these methods can be used to process multiple tissue samples at the same time, which can further improve the diagnostic efficiency of clinical infectious diseases.

This study differs from other studies in that it provides a more comprehensive assessment of different currently reported pretreatment methods. In this study, it was impossible to obtain sufficient tissues from a single patients to established infectious model in vitro [11], thus, fresh pork was used to established different infectious tissues models, which simulated tissues samples collected from PJI patients to a large extent. To reduce the possibility of contamination, the pork cubes were immersed in PBS containing antibodies (penicillin, streptomycin and amphotericin B) for 1 h before further experiment. Thereafter, tissue samples collected from PJI cases were pretreated by different methods used in pork tissues to further verify the effects of different methods on microbial yield.

To study the effects of different pretreatment methods on different microbial species, *S. aureus*, *E. coli*, and *C. albicans* were chosen as representatives of gram-positive, gram-negative bacteria, and fungi to construct infectious tissues models in vitro. The concentration used in single-surface infection was based on previous reports to avoided massive bacterial growth and while convenient for CFUs counting [12].

There are still some issues about optimal pretreatment methods on tissues, because insufficient pretreatment might result in low bacteria yield released from tissues, while excessive pretreatment might reduce bacterial viability. Mohamed Askar reported that the use of mechanized steel ball grinding and homogenization might reduce the recovery of bacteria from tissue samples [6]. However, this study showed there were no significant effects on the viability of bacteria pretreated by various methods. This might be attributed to the usage of precooled working solution for various pretreatment, which could reduce heat generation during processing and thereby avoid reduce of bacterial vitality.

This study found that the average CFUs obtained from tissues pretreated by MH in every model were higher than that pretreated by DC and MM, which is basically consistent with the results of Sylvio Redanz et al [11]. The DTT and SF were also performed in this study because previous reported that the DTT can homogenize tissues and facilitate microorganisms release from tissue samples [7], and the application of SF was reported to break the biofilm on the surface of a prosthesis and thus promote bacteria release [13–16]. And we showed that DTT’s ability to facilitate bacteria release from infectious tissues was similar to that of MH, while SF’s ability to promote bacteria release from tissues was not as good as that of MH and DTT. In the internal infection model, only MH could facilitate bacteria release, and DTT did not show this role, which was similar to MM and SF. All above results demonstrated that MH had the best effect on high microbial yield from tissues of various models.

Finally, tissue specimens collected from PJI patients who was diagnosed according to MSIS criteria were pretreated by MH, MM, DTT, SF and DC. And the results were consistent with the infectious pork tissues. The number of colonies obtained from tissues pretreated by MH was significantly higher than that pretreated by other methods, which further confirms the superiority of MH under clinical conditions. This was also consistent with the reports of Mohamed Askar and Sylvio Redanz [6, 11].

It is worth noting that tissue sample processing takes longer time and more tedious than synovial fluid, false positive induced during this process were also a concern. This study showed that there were no false positive culture results in the negative control samples pretreat by MH and DTT. The reason might be that, when tissues were pretreated by MH and DTT, the samples were basically sealed during the whole process, while samples were exposed to the air when pretreated by MM and thus contaminations might be induced.

There were some limitations in this study: 1) This study constructed infectious tissues model in vitro only by *S. aureus*, *E. coli*, and *C. albicans*, while other pathogens would also include in PJI, such as *Mycobacterium tuberculosis* and non-tuberculous mycobacteria, mycoplasma, etc.; 2) The purpose of this study was to evaluate the effect of different pretreatment methods on microbial yield, but it was impossible to determine their actual diagnostic efficiency in clinical practice, further investigation should be performed; 3) The study design was transversal, so it was insufficient to measure the effect. Further longitudinal study comparing the pre and post application steps of the proposed methods should be performed.

## Conclusion

Mechanical homogenization and dithiothreitol could significantly facilitate bacteria released from tissues than sonificated, manual milling, and direct culture methods, which were recommended for tissues sample pretreatment in clinical, and potential improve the diagnostic efficiency of infectious disease.

## Supplementary Information

**Additional file 1: Table 1.** Microbial yield recovered from three infection models by various pretreatment methods.

## Data Availability

The datasets used and/or analysed during the current study are available from the corresponding author on reasonable request.

## Abbreviations

SA: *Staphylococcus aureus*
EC: *Escherichia coli*
CA: *Candida albicans*
MM: Manual milling
MH: Mechanical homogenization
SF: Sonificated
DTT: Dithiothreitol
DC: Direct culture
ATCC: American Type Culture Collection
PJI: Periprosthetic joint infection
CFU: Colony-forming units
MSIS: The American Society for Musculoskeletal Infection
