# Supplementary material for: Microbial yield from infectious tissues pretreated by various methods: an invitro study
Source: BMC Musculoskelet Disord. 2021 Feb 21;22:209. doi: 10.1186/s12891-021-04071-5 (PMC7898421; doi:10.1186/s12891-021-04071-5)
Supplement: Supplementary file 1 — Additional file 1: Table 1. Microbial yield recovered from three infection models by various pretreatment methods. [file 12891_2021_4071_MOESM1_ESM.docx]

**Table 1 Microbial yield recovered from three infection models by various pretreatment methods.**

| **1.Microbial yield recovered from single-surface infection models by various pretreatment methods.** | | | |
| --- | --- | --- | --- |
| Microorganisms | Pretreatment methods | CFU/ml (95% CI) | *P*- value |
| *S. aureus* | MM | 611 ± 101 (538-683) | *P* <0.05 |
|  | MH | 999 ± 141(898- 1100) |  |
|  | SF | 609 ± 96 (540-679) |  |
|  | DTT | 938 ± 136 (840-1035) |  |
|  | DC | 533 ± 108 (455-611) |  |
| *E. coli* | MM | 451 ± 192 (313-588) | *P* <0.01 |
|  | MH | 1373 ± 132 (1278-1468) |  |
|  | SF | 480 ± 135 (383-576) |  |
|  | DTT | 1347 ± 162 (1231-1463) |  |
|  | DC | 393 ± 178 (265-520) |  |
| *C. albicans* | MM | 440 ± 70 (391-488) | *P* <0.05 |
|  | MH | 818 ± 122 (730-905) |  |
|  | SF | 435 ± 68 (386-484) |  |
|  | DTT | 771 ± 108 (694-849) |  |
|  | DC | 393 ± 178 (298-421) |  |
| **2. Microbial yield recovered from full-surface infection models by various pretreatment methods.** | | | |
| Microorganisms | Pretreatment methods | CFU/ml (95% CI) | *P*- value |
| *S. aureus* | MM | 4014 ± 888 (3378-4650) | *P* <0.05 |
|  | MH | 6268 ± 1019 (5539-6997) |  |
|  | SF | 3960 ± 875 (3334-4568) |  |
|  | DTT | 6227 ± 1000 (5512-6942) |  |
|  | DC | 3255 ± 1048 (2505-4005) |  |
| *E. coli* | MM | 3079 ± 934 (2410-3747) | *P* <0.05 |
|  | MH | 7058 ± 920 (6400-7717) |  |
|  | SF | 3060 ± 902 (2415-3706) |  |
|  | DTT | 6853 ± 858 (6239-7466) |  |
|  | DC | 2533 ± 637 (2077-2989) |  |
| *C. albicans* | MM | 3080 ± 842 (2477-3682) | *P* <0.05 |
|  | MH | 5274 ± 966 (4583-5966) |  |
|  | SF | 2971 ± 890 (2334-3608) |  |
|  | DTT | 5269 ± 853 (4658-5879) |  |
|  | DC | 2287 ± 851 (1678-2896) |  |
| **3. Microbial biomass recovered from internal infection models by various pretreatment methods.** | | | |
| Microorganisms | Pretreatment methods | CFU/ml (95% CI) | *P*- value |
| *S. aureus* | MM | 267 ± 68 (218-316) | *P* <0.01 |
|  | MH | 636 ± 90 (571-701) |  |
|  | SF | 300 ± 73 (247-353) |  |
|  | DTT | 270 ± 63 (224-315) |  |
|  | DC | 208 ± 95 (140-277) |  |
| *E. coli* | MM | 313 ± 78 (257-369) | *P* <0.01 |
|  | MH | 698 ± 134 (601-794) |  |
|  | SF | 298 ± 72 (246-351) |  |
|  | DTT | 306 ± 93 (239-373) |  |
|  | DC | 234 ± 82 (175-293) |  |
| *C. albicans* | MM | 169 ± 60 (125-212) | *P* <0.01 |
|  | MH | 359 ± 55 (319-399) |  |
|  | SF | 164 ± 58 (122-206),) |  |
|  | DTT | 172 ± 58 (130-213) |  |
|  | DC | 119 ± 47 (85-154) |  |
